# Improving research transparency: An interpretation of the updated consolidated standards of reporting trials 2025 guideline from the perspective of clinical trials in oncology^☆^

**DOI:** 10.1016/j.cpt.2025.07.002

**Published:** 2025-07-08

**Authors:** Yaguang Peng, Peng Lyu, Xiaoxia Peng

**Affiliations:** a Center for Clinical Epidemiology & Evidence-based Medicine, National Center for Children's Health, Beijing Children's Hospital, Capital Medical University, Beijing 100045, China; b Cancer Pathogenesis and Therapy, Chinese Medical Association Publishing House, Beijing 100052, China; c Key Laboratory of Knowledge Mining and Service for Medical Journals, National Press and Publication Administration, Beijing 100052, China

## Introduction

Well-designed, strictly implemented, and fully standardized randomized controlled trials (RCTs) are a prerequisite for developing reliable scientific evidence, which can improve clinical practice, health outcomes, and ultimately benefit patients. Suboptimal reporting is pervasive in medical research, resulting in biased research records and persistent uncertainty about the quality of available evidence.^1^, ^2^, ^3^, ^4^

The standardization of research reports has attracted considerable attention. In 1996, the Consolidated Standards of Reporting Trials (CONSORT) was first published to improve the quality of RCTs and enhance the reproducibility of trial methods, results, and inferences.^5^ CONSORT provides important guidelines for reviewers, editors, and readers to assess the reliability and authenticity of study findings and has been widely praised in practical applications.^6^ Since then, CONSORT has been revised three times: in 2001,^7^ 2010,^8^ and the current update in 2025.^4^ The CONSORT group has worked tirelessly to promote standardized and transparent clinical trial reporting.^9^ With the CONSORT statement, clinical trial reporting has improved significantly, featuring structured and transparent documentation instead of arbitrary inference, balanced emphasis on both process and outcomes (rather than being result-oriented), and integration of isolated studies into credible scientific evidence. During these changes, CONSORT made significant contributions and played a leading and exemplary role in the standardization of clinical research reports for other clinical study designs.

The development of antitumor drugs is closely intertwined with high-quality clinical trials, making it a global research focus. With the rapid development of related basic and clinical research recently, many clinical oncology studies have been conducted, and remarkable advances have been made in the research and development of antitumor drugs in China.^10^ As of 2020, approximately one-third of global clinical trials have been conducted in China, and this proportion has increased significantly, especially for oncology clinical trials.^11^^,^^12^ However, oncology trials are characterized by unique challenges in their design and implementation. First, the complexity of the trial design arises from the need to integrate adaptive designs, multistage protocols, and crossover treatment strategies, while balancing the validation of composite endpoints, surrogate endpoints, and survival benefits. Second, significant heterogeneity in patient populations and disease pathogenesis and progression, such as variations in tumor subtypes, staging, and molecular profiles, complicate the prediction of therapeutic responses, necessitating biomarker-driven stratification to enhance precision. Ethically, researchers must navigate the “therapeutic desert” faced by terminally ill patients, dynamically weighing drug toxicity, quality of life, and potential clinical benefits, with particular emphasis on ensuring transparent informed consent and rigorous risk disclosure. The recently updated CONSORT 2025 statement reinforces standardized reporting requirements for complex interventions, patient subgroup analyses, and adverse event grading with the aim of ensuring transparent and reliable evidence. Considering the distinct features of oncology research, a context-specific interpretation of the CONSORT guidelines is imperative. These considerations demand that researchers adhere to evidence-based frameworks and develop oncology-specific methodological reflections, ultimately fostering the synergistic advancement of ethical rigor and scientific validity in clinical research. Using the updated CONSORT 2025 statement, this article outlines the limitations and challenges faced in oncology clinical trials and explores adaptive solutions.

## Main changes in consolidated standards of reporting trials 2025

CONSORT 2025 added seven new items, revised three items, deleted one, and integrated and restructured some items based on the 2010 version [Table 1].

**Table 1:** Interpretation of main changes in Consolidated Standards of Reporting Trials 2025 compared to the 2010 version.

Changes type	CONSORT 2025 item	Interpretation	Oncology-specific examples
Addition	4	Clarify the data shared to enhance repeatability, including where and how individual de-identified data were collected, definitions of variables, analysis codes, and materials related to the intervention. If sharing is not planned, it should be clearly stated and explained.	• Share genomic raw data (e.g., FASTQ files) and variant calling codes for biomarker-driven trials • Provide protocols for liquid biopsy ctDNA analysis
	5b	Emphasize the conflict of interest statements of all authors. Enhance the credibility of the research.	• Disclose pharma funding for targeted therapy trials • Report institutional patents on CAR-T technologies
	8	Expect that patients or the public can form partnerships with researchers, involving the study design and conduct, and report, interpret, and disseminate the trial. The patients or the public are not participants in the trial. They can be individuals who currently or have had certain health conditions, as well as their family members, caregivers, social workers, and other members of the public. The participation of patients and the public is conducive to improving the efficiency and acceptability of recruitment and follow-up, selecting the outcomes and their measurements that the target population truly needs, and improving communication among participants and the dissemination of results.	• Involve cancer survivors in designing PROs for immunotherapy QoL assessment • Engage caregivers in defining toxicity reporting thresholds
	12b	Consider the influence of the provider conditions of the interventions on the effect. Not all studies need to report the qualification criteria of the intervention provider. When the outcome of the intervention is highly associated with the characteristics of the intervention provider, such as human resources, technical levels, and management, the therapeutic effect will be affected.	• Specify CAR-T cell manufacturing facility certifications (e.g., GMP compliance) • Detail radiologist experience thresholds for RECIST assessments
	15	Adverse events can include pre-considered and non-pre-considered adverse events. The former can be achieved through active monitoring and systematic assessment, while the latter is more of a passive report. For different types of adverse events, their definitions, measurement variables, participant-level analysis indicators, summary measurement values for each group, and the analysis time windows should be reported. A preferred approach is to obtain the individual data. When conducting a summary analysis, the process of classifying adverse events should be described, and the severity, grade, involved system, and reasons for withdrawal from the trial due to adverse events should be defined.	• Define CRS grading per the ASTCT criteria in CAR-T trials • Report neurotoxicity onset timelines in bispecific antibody trials
	21b, 21c	Emphasize the reports of different analytical populations and the methods for handling missing values to reduce the risk of data manipulation. ITT analysis is the key to controlling bias in RCTs. The outcome data missing due to loss to follow-up or missing results, or due to poor compliance, even if filled in and efforts have been made to conduct “ITT,” are not strictly ITT analyses. The handling of missing data also needs to be fully reported, including the number, proportion, and reasons for the missing data, comparison with the observed data characteristics, whether interpolation was adopted, the specific interpolation method, and more importantly, the results of sensitivity analysis, and the impact of missing data on the interpretation of the results. At least a summary of the sensitivity analysis of the missing data should be reported. The detailed results should be given in the supplementary materials.	• Sensitivity analysis for OS data with high dropout in metastatic trials • Multiple imputation for missing biomarker data in neoadjuvant studies
	24	The intervention taken during the trial might differ slightly from that at the design stage, which may be due to executive ability, compliance, intervention diversity, and complexity, etc. Concomitant treatments outside the trial intervention might introduce post-randomization bias, especially when the concomitant treatments are significantly associated with the primary outcome, having a greater impact on the reliability of the results. It is necessary to fully report the considerations at the design stage, the disparity from the design stage, the type and frequency of the accompanying treatment, and, more importantly, the differences between groups.	• Report deviations in radiotherapy dosimetry • Document concomitant use of antifungals during AML chemotherapy
Revision	3	Emphasize access to pre-planned SAP. Before the participant allocation, data collection, or dataset for analysis, any change in the trial protocol and SAP due to unexpected challenges or the emergence of new evidence should be reviewed and signed.	• Pre-specify subgroup analyses for PD-L1 expression levels • Lock SAP before the interim analysis of PFS endpoints
	10	Reports on un-pre-specified outcomes or analyses. The change of the plan would affect the trial design and conduct.	• Flag post-hoc analyses of TMB predictive value • Justify added endpoints like MRD negativity in protocol amendments
	26	The number of participants with available data at each time point for each primary and secondary outcome was emphasized. If possible, the reasons for the unavailability of data should be reported, such as loss to follow-up or censoring. The relative effects (hazard ratio or odds ratio) and absolute effects (risk difference) for the binary primary outcome should be reported to meet the needs of different readers.	• Report attrition rates at 24-month OS follow-up • Provide reasons for missing RECIST assessments (e.g., COVID-19 scan delays)
Deletion	30	Generalizability was deleted and incorporated into limitations item 30. If the report fails to effectively reflect the internal validity of the trial, it would indicate the low quality of the trial. At this point, it seems insignificant to talk about generalizability.	• Discuss applicability of basket trial results to rare subtypes (e.g., NTRK fusions)
Integration and restructuring	Safety/adverse events 7, 15, 21a, 23a, 27	Integrated reporting on how harms were assessed and analyzed.	• Unified reporting of irAEs across CTCAE categories
	14, 26	Integrated reporting on how outcomes were measured and analyzed	
	24	Added reporting about the intervention administered (refer to above).	
	Open science 2–5	Restructure the open science section. In addition to the traditional trial registration and open access of protocols, data sharing of de-identified individual participants and statistical codes have also been advocated, providing the possibility of achieving aggregated analysis based on individual data and the possibility of statistical analysis of review data.	
	All	Aligned terms in CONSORT with SPIRIT checklist.	

AEs: Adverse events; AML: Acute myeloid leukemia; ASTCT: American Society for Transplantation and Cellular Therapy; CAR-T: Chimeric antigen receptor-therapy; CONSORT: Consolidated Standards of Reporting Trials; COVID-19: Coronavirus disease 2019; CRS: Cytokine release syndrome; CTCAE: Common terminology criteria for adverse events; ctDNA: Circulating tumor DNA; FASTQ: The most commonly used file format for storing nucleotide sequence information or protein sequence information; GMP: Good manufacturing practice; irAEs: Immune-related AEs; ITT: Intention-to-treat; MRD: Measurable residual disease; NTRK: NeuroTrophin Receptor Kinase; OS: Overall survival; PD-L1: Programmed death-ligand 1; PFS: Progression-free survival; PROs: Patient-reported outcomes; QoL: Quality of life; RCTs: Randomized controlled trials; RECIST: Response evaluation criteria in solid tumors; SAP: Statistical analysis plan; SPIRIT: Standard Protocol Items: Recommendations for Interventional Trials; TMB: Tumor mutational burden.

The updated CONSORT checklist and detailed item descriptions are available on its official website (https://www.consort-spirit.org). More detailed examples and interpretations can be obtained, providing convenience for understanding the list of items. Detailed interpretations of each item have been published in Chinese^9^ and were not elaborated here. Based on the CONSORT 2025, we discuss the challenges and considerations in reporting clinical trials in oncology and provide suggestions from different perspectives.

## Applicability and challenges with consolidated standards of reporting trials 2025 in oncology research

The CONSORT statement is a general guideline for reporting RCTs and provides a minimum set of recommendations for reporting trial details. Many CONSORT extensions have been developed to address the methodological issues of reporting different trial designs, data, and interventions, including recommendations for adaptive designs,^13^ early phase trials^14^ multi-arm trials,^15^ n-of-1 trials,^16^ and patient-reported outcomes, among others.^17^

When the CONSORT 2025 statement is applied to an oncology clinical trial, the updated items enable readers to gain a clearer understanding of the trial process and improve the study's transparency. For instance, the data sharing in item 4 can promote data transparency, such as liquid biopsy and gene sequencing involved in tumor-related trials. Patients and public participation in item 8 will enhance the consideration of patients' feedback on individualized therapy (such as chimeric antigen receptor T-cell therapy [CAR-T]), to reflect the development trend of “patient-oriented.” The harm statement in item 15 requires systematic reporting of unique safety events during immunotherapy (such as cytokine storms). However, significant reporting gaps persist in oncological clinical trials, requiring urgent revision beyond the current CONSORT framework. For instance, studies highlight critical deficiencies: a recent analysis of targeted therapy trials revealed that 67% inadequately reported biomarker validation methods, which are crucial for interpreting results in precision oncology.^18^ These specific gaps, stemming from the unique characteristics of oncology research outlined above (e.g., biomarker-driven designs, complex interventions, heterogeneous endpoints), underscore the limitations of existing generic or non-oncology-specific extensions in fully addressing the reporting needs of this field. In the section below, we summarize the following six aspects and discuss the issues that need to be considered and reported in oncology trials.

### Selection of biomarkers

Biomarker-driven designs have been applied in oncological clinical trials, including screening and enrichment strategies. There is a lack of reporting standards for targeted therapy in patients undergoing gene mutation screening. Key information, including biological and clinical rationales, biomarker prevalence, safety data, biomarker validation methods, overall trial design, and subgroup efficacy characterization, should be reported in detail. Currently, CONSORT and its corresponding extensions do not include relevant requirements regarding the selection of biomarkers, the detection process, or the interpretation of results. To address this gap, we propose the following minimum reporting standards for biomarker-driven oncology trials [Supplementary Table 1].

### Complexity of combination therapy

Combination or integrated therapy is currently a popular topic and a future development trend. The antitumor mechanisms of different treatment strategies vary; however, there may also be synergistic or antagonistic effects among intervention mechanisms. In clinical trials involving multiple intervention mechanisms, the interactions between different interventions should be analyzed and reported extensively to ensure clearer causal inferences. Ancillary analyses in item 28 seemed to include an interaction analysis. Additionally, the theoretical basis of specific combined or integrated therapies should be reported in detail.

### Implementation and safety of complex interventions

Recently, immunotherapy with complex processes has conflicted with standardized interventions in classic RCTs. For example, CAR-T treatment involves cell extraction, preparation, and reinfusion.

Gene therapy may require interventions at multiple stages, such as viral vector delivery and gene editing. Operation and quality control at each step may affect the outcomes. The implementation of such detailed interventions and quality control is particularly important for the interpretation of trial results. For more complex interventions, CONSORT already has corresponding principles, namely, the detailed elaboration of the intervention implementation in item 24. Individualized intervention implementation and corresponding quality control measures should be described in detail.

### Selection of outcome endpoints for highly heterogeneous tumors

Item 14 clearly distinguishes between primary and secondary outcomes and explains clinical rationality. However, the main outcomes of efficacy evaluations in oncology clinical trials are heterogeneous. For example, some solid tumors use progression-free survival (PFS) as the primary endpoint, whereas for hematological tumors, more attention may be paid to the complete response rate (CR). Domestic and foreign regulatory authorities have recently issued corresponding guiding principles for the use of measurable residual disease (MRD) to be used as a surrogate for specific tumors. In this regard, on the basis of the following relevant principles, the basis for outcome selection should also be clearly stated in the report.

### Standardized application of emerging technologies

Many emerging technologies, including liquid biopsy, artificial intelligence-assisted diagnosis, and methods such as deep learning algorithms or recurrence and composite outcome analysis, have emerged; however, the CONSORT 2025 statement has limited coverage. Using the CONSORT framework, the envisioned reporting statement would include several additional aspects, as detailed in Supplementary Table 2.

### Ethics and social equity

Some innovative therapies, such as CAR-T cell and gene therapies, have relatively high costs and technical thresholds. There may also be differences in regional accessibility. Economic feasibility and social equity may lead to biases and affect trial results. More importantly, ethical risks may also be associated with accessibility or fairness. Consequently, we suggest that discussions on economic burden and fairness should be reported. It can also be combined with the CONSORT Equity Extension to report on resource allocation and patient representation.

Additionally, oncological clinical trials using a single-arm design and/or with small sample sizes should focus on transparent reporting. Although domestic and foreign regulatory authorities have issued relevant guiding principles for single-arm designs, the corresponding reporting guidelines or extensions have not yet been unified. Thus, the premise for adopting a single-arm design must be clarified. The problem of small sample sizes in trials occurs due to rare diseases or the high economic costs of the intervention. For example, due to the high cost and technical difficulty of gene therapy, the sample size of early trials is often small, which may lead to insufficient statistical power. Adverse reactions to certain gene therapies may be extremely rare, and common statistical methods may not be appropriate. Appropriate statistical analysis methods must be planned for the statistical analysis plan (SAP) in advance. This also emphasizes cooperation between statistical experts and investigators throughout the clinical trial process.

## Considerations and implementation suggestions from different users

It should be emphasized that CONSORT is not a quality assessment tool. However, clear and transparent reporting of trial protocols can promote good trial design and implementation, reveal research flaws, and enable readers to better understand the validity of the results and conclusions. Readers, peer reviewers, clinicians, guideline writers, patients, the public, and editors can also use CONSORT 2025 to appraise the reporting of randomized trials.^4^ Different users may have different foci when applying CONSORT, especially when there is no expansion for oncology clinical trials. It is recommended that journals mandate the submission of a completed CONSORT 2025 checklist alongside manuscripts and that trial registries incorporate data-sharing plans in their registration fields.

First, if there is a corresponding extended list, it can be used directly to report the trial results. For example, in the CONSORT-adverse drug event (ADE) for adaptive design, for those without an expanded list, it is suggested that under the CONSORT framework, relevant issues should be clarified as fully as possible to enhance research transparency. For example, in biomarker-driven clinical trials, the validation of biomarkers is recommended. For trials involving genetic testing, the availability of original data from gene sequencing and the analysis code should be fully elaborated upon during data sharing.

The CONSORT list provides a tool for evaluating manuscripts, including the transparency and completeness of the trial. For research involving combined therapy or integrated therapy of tumors or some relatively complex interventions, more attention needs to be paid to complex designs, including the design of trials and interventions, and the interactions among interventions should also be considered. For emerging technologies such as liquid biopsy, the transparency of interventions, methods, and implementation is even more crucial.

For journals and editors, the submission guidelines should be updated in a timely manner, clearly requiring authors to submit the complete CONSORT 2025 list. We encourage authors to publicly disclose trial data and analysis codes to promote open science practices. We set up a special column to promote the popularization and application of reporting standardization, and the development of reporting guidelines specified for oncological clinical trials. For a journal article of several thousand words, considering the length of the article, it is unrealistic to include all items related to the statistical methods in the main text. However, journals can request relevant links, so that interest holders can obtain detailed statistical analysis methods, plans, and codes. This also applies to interventions. A complete report should not be confined to a few pages of a journal.

Finally, to ensure widespread comprehension and effective utilization of the reporting standardization tools, structured training programs on CONSORT 2025 should be implemented for the diverse stakeholders involved in oncology trials. The CONSORT Group, potentially in collaboration with major oncology societies or journals, should develop standardized training modules (e.g., online courses, webinars, downloadable resources) covering the core principles and specific extensions relevant to oncology. Institutions, contract research organizations (CROs), and regional consortia should then conduct targeted workshops and hands-on sessions tailored to specific roles (e.g., investigators, research coordinators, data managers, statisticians, and medical writers) to facilitate practical applications.

## Future direction and appeal

It is important to emphasize again that the CONSORT statement provides a basic framework for reporting clinical trials, constituting the minimum requirement to ensure clarity, transparency, and ultimately, the reflection of the authenticity and reliability of the trial design, implementation, and analysis. High-quality reporting is a fundamental prerequisite for reproducibility.^19^ Given the persistent and critical reporting gaps identified in oncology trials, coupled with the field's unique methodological and practical complexities (e.g., biomarker stratification, combination therapies with potential interactions, complex interventions such as cellular therapies, diverse endpoint selection, and emerging technologies), the development of a dedicated CONSORT extension for oncological clinical trials (CONSORT-Oncology) is not only warranted but urgently needed. Such an extension could provide tailored guidance for addressing the specific challenges outlined in this study. For instance, in biomarker-driven trials, details should be provided regarding the basis for biomarker selection (from a clinical and/or biological rationale), validation methods (such as sequencing platform certification), and result interpretation (such as false-positive rate controls). Regarding the adoption of combined or integrated therapies, interactions among interventions should have been considered and reflected in statistical analysis methods and results. Furthermore, when applying emerging technologies, it is necessary to disclose the technical details of detection (such as the sensitivity of circulating tumor DNA [ctDNA] detection, sequencing depth), training, and verification processes of the artificial intelligence (AI) model prediction.

Extending equity is crucial for ethics and social equity. Consider including extended items similar to “Economic Burden and Resource Allocation,” which necessitate an analysis of differences in treatment costs, insurance coverage, and regional accessibility. For instance, disparities in access to oncology clinical trials are well-documented and significantly impact equitable care. Studies have shown that factors such as socioeconomic status, geographic location (e.g., distance from major academic centers), race/ethnicity, and insurance type create substantial barriers to trial participation.^20^^,^^21^ Integrating specific metrics related to these dimensions, such as the reporting of patient demographics (including zone improvement plan code or region), insurance status, and trial enrollment rates stratified by relevant factors, would strengthen reporting standards and directly address the equity concerns raised by high-cost therapies such as CAR-T.

Finally, through interdisciplinary collaboration with experts in bioinformatics and ethics, the framework of technical standardization and ethical reporting can be improved. Through the coordination of academic societies and associations, experts from various fields are required to discuss or adopt corresponding scientific methods, conduct research, form specific supplementary items or extended versions, and promote the establishment of a clinical research data sharing platform in oncological research, facilitating transparent research and open scientific practice. Through the joint efforts of researchers, journals, and guideline makers, a balance between standardization and innovation in high-quality oncological clinical trial reports can be achieved.

## Authors contribution

Yaguang Peng: Information collection and data curation, analysis, and writing original draft; Peng Lyu: Project administration and writing-review & editing; Xiaoxia Peng: Conceptualization and methodology, and writing-review & editing. All the authors have read and approved the final paper.

## Ethics statement

None.

## Data availability statement

The datasets used in the current study are available from the corresponding author on reasonable request.

## Declaration of generative AI and AI-assisted technologies in the writing process

The authors declare that generative artificial intelligence (AI) and AI assisted technologies were not used in the writing process or any other process during the preparation of this manuscript.

## Funding

This work was supported by the Talent Development Plan for High-level Public Health Technical Personnel Project in Beijing, Beijing Municipal Health Commission [No. XKGG-02-03].

## Conflict of interest

The authors declare that they have no known competing financial interests or personal relationships that could have appeared to influence the work reported in this paper.
