# Supplementary material for: Improving research transparency: An interpretation of the updated consolidated standards of reporting trials 2025 guideline from the perspective of clinical trials in oncology
Source: Cancer Pathog Ther. 2025 Jul 8;4(1):75–80. doi: 10.1016/j.cpt.2025.07.002 (PMC12834674; doi:10.1016/j.cpt.2025.07.002)
Supplement: Multimedia component 1 [file mmc1.docx]

**Appendix A. Supplementary data**

**Supplementary Table 1:** Reporting issues in biomarker-driven oncology trials.

| Reporting domain | Essential elements |
| --- | --- |
| Biomarker rationale | • Biological mechanism linking biomarker to therapy  • Clinical evidence (e.g., phase II data) |
| Analytical validation | • Sensitivity/specificity rates  • False positive/negative rates  • Platform certification  • Sample quality control criteria (e.g., tumor content ≥20%) |
| Result interpretation | • Predefined positivity thresholds (e.g., PD-L1≥50%)  • Statistical adjustment for multiple testing  • Handling of equivocal results |
| Clinical utility | • Positive/negative predictive values  • Subgroup efficacy analysis (e.g., forest plots by biomarker status) |

PD-L1: Programmed death-ligand 1.

**Supplementary Table 2:** Reporting issues for emerging technologies.

| Technology | CONSORT 2025 Item | Oncology-specific extension |
| --- | --- | --- |
| Liquid biopsy | Item 14 | • Report ctDNA assay sensitivity/LoD  • Detail sample processing protocols  • Specify variant calling pipeline |
| AI algorithms | Item 5b | • Disclose training data demographics  • Provide algorithm code repository  • Document version freeze date  • Handling of equivocal results |
| Composite endpoints | Item 6b | • Define component weight justification  • Publish adjudication charter  • Report sensitivity analyses |

Note: These are only preliminary ideas and should be further improved by reaching a consensus among the expert group. AI: Artificial intelligence; CONSORT: Consolidated Standards of Reporting Trials; ctDNA: Circulating tumor DNA; LoD: Limit of detection
